# Aptamer-Based Fluorescence Quenching Approach for Detection of Aflatoxin M_1_ in Milk

**DOI:** 10.3389/fchem.2021.653869

**Published:** 2021-03-24

**Authors:** Qinqin Qiao, Xiaodong Guo, Fang Wen, Lu Chen, Qingbiao Xu, Nan Zheng, Jianbo Cheng, Xiuheng Xue, Jiaqi Wang

**Affiliations:** ^1^ Laboratory of Quality and Safety Risk Assessment for Dairy Products of Ministry of Agriculture and Rural Affairs, Institute of Animal Science, Chinese Academy of Agricultural Sciences, Beijing, China; ^2^ College of Information Engineering, Fuyang Normal University, Fuyang, China; ^3^ Anhui Agricultural University, Hefei, China; ^4^ School of Agriculture and Biology, Shanghai Jiao Tong University, Shanghai, China; ^5^ Milk and Dairy Product Inspection Center of Ministry of Agriculture and Rural Affairs, Beijing, China; ^6^ College of Animal Sciences and Technology, Huazhong Agricultural University, Wuhan, China

**Keywords:** aflatoxin M1, aptasensor, fluorescence, quenching, food safety

## Abstract

Aflatoxin M_1_ (AFM_1_), one of the most toxic mycotoxins, is a feed and food contaminant of global concern. In this study, we developed a fast and simple method for detection of AFM_1_ based on a structure-switching signaling aptamer. This aptasensor is based on the change in fluorescence signal due to formation of an AFM_1_/aptamer complex. To generate the aptasensor, the specific aptamer was modified with FAM (carboxyfluorescein), and their complementary DNAs (cDNA) were modified with a carboxytetramethylrhodamine (TAMRA) quenching group. In the absence of AFM_1_, the aptamers were hybridized with cDNA, resulting in quenching of the aptamer fluorescence due to the proximity of the aptamer’s fluorophore to the quenching group on the cDNA. On the other hand, in the presence of AFM_1_, a structural switch in the aptamer was induced by formation of an AFM_1_/aptamer complex. Changes in the structure of the aptamer led to the release of the cDNA, causing the generation of a fluorescence signal. Thus, AFM_1_ concentrations could be quantitatively monitored based on the changes in fluorescences. Under optimized conditions, this assay exhibited a linear response to AFM_1_ in the range of 1–100 ng/mL and a limit of detection of 0.5 ng/mL was calculated. This proposed aptasensor was applied to milk samples spiked with a dilution series of AFM_1_, yielding satisfactory recoveries from 93.4 to 101.3%. These results demonstrated that this detection technique could be useful for high-throughput and quantitative determination of mycotoxin levels in milk and dairy products.

## Introduction

Mycotoxins are highly toxic secondary metabolites and produced by several fungal genera, including *Aspergillus*, *Fusarium*, and *Penicillium* (Li et al., 2012; Liu et al., 2015). To date, more than 400 mycotoxins have been identified (Selvaraj et al., 2015). Among them, aflatoxins (AFs), deoxynivalenol (DON), ochratoxin (OTA), fumonisin B_1_ (FB_1_), zearalenone (ZEN), and T-2 toxin (a trichothecene mycotoxin) are common mycotoxins (Yang et al., 2017). As the most toxic AF, aflatoxin B_1_ (AFB_1_) is classified as a group 1 carcinogen by the International Agency for Research on *Cancer* (IARC) (Iqbal et al., 2015). In addition, aflatoxin M_1_ (AFM_1_) is a hydroxylated metabolite of AFB_1_, and approximately 2–6% of AFB_1_ in contaminated animal feed is converted to AFM_1_ in milk (Abera et al., 2019). AFM_1_ mainly causes chronic hepatitis and liver tumors in humans, which was classified as group 2 and changed to group 1 carcinogen by IARC (Karczmarczyk et al., 2016). Due to the serious harm caused to human and animal health by mycotoxins, many countries and international organizations (e.g., the European Union and the Joint FAO/WHO Expert Committee on Food Additives) have established relevant standards for limiting AFM_1_ contamination in foods to extremely low levels (Wang et al., 2013; Song et al., 2014). In Europe, the maximum residue level of AFM_1_ in milk is 0.05 μg/kg (European Commission, 2006). In China and the United States, the maximum tolerated level for AFM_1_ in milk is 0.5 μg/kg (Busman et al., 2015; Mao et al., 2015). Once raw milk is contaminated with AFM_1_, conventional heat treatment (pasteurization or ultra-high temperature) for milk and dairy products is not easy to degrade AFM_1_ (Beitollahi et al., 2020).

Despite strict regulation and control of AFM_1_ levels in milk, AFM_1_ pollution still occurs frequently (Li et al., 2017; Zheng et al., 2017). Consequently, methods for detecting AFM_1_ in milk are attracting increasing public attention. Many methods for detecting AFM_1_ have been developed, including high-performance liquid chromatography (HPLC) (Mao et al., 2015; Pietri et al., 2016), enzyme-linked immunosorbent assay (ELISA) (Li et al., 2009; Kav et al., 2011), immunochromatography (Busman et al., 2015), thin-layer chromatography (TLC) (Sassahara et al., 2005), and liquid chromatography coupled with mass spectroscopy (LC-MS)(Vaclavik et al., 2010). However, instrument-based analytical methods suffer from several drawbacks, including the requirements of technical expertize and complicated, time-consuming sample pretreatment, making these approaches unsuitable for field operation(Eivazzadeh et al., 2016). Moreover, antibody-based immunoassay systems require high-quality antibodies, which are often too expensive, unstable, or susceptible to degradation and denaturation for field applications(Istamboulia et al., 2016). In the past few years, several groups of researchers have pursued AFM_1_ detection technology on immunoassays combined with fluorescence (Shen et al., 2017), electrochemistry (Parker and Tothill, 2009) or chemiluminescence (Vdovenko et al., 2014). Biosensor-based aptamers have attracted increasing attention due to their numerous advantages, including high sensitivity and specificity, easy synthesis and modification, labeling, reusability, and portability (Rhouati et al., 2016). Malhotra et al. used laminated magnetic beads to select this AFM_1_ aptamer by exponential enrichment (SELEX technique). The aptamer has a unique structure that encompasses two overlapping stem loops without conserved motif or G-quadruplex, which has lowest dissociation constant value (K_d_ = 35 nM). The researchers confirmed that the aptamer has good affinity against AFM_1_. In addition, the specific aptamer has previously been applied for AFM_1_ sensing for our research team (Malhotra et al., 2014). Guo X et al. developed a biosensor based on this aptamer to detect AFM_1_ and combined the advantages of strong recognition ability of the aptamer to AFM_1_ and excellent amplification efficiency of the RT-qPCR technique to improve sensitivity (Guo et al., 2016). Recently, Kordasht H et al. used cyclic voltammetry and differential pulse voltammetry techniques to a bioassay based on this aptamer for the determination of AFM_1_ (Kordasht et al., 2019).

In particular, structure-switching fluorescence-quenching signal adaptation platforms are considered promising approaches for detection of various biomolecules due to their high sensitivity and specificity, and the fluorescence-quenching approach has been used for the detection of AFB_1_ in our previous research(Chen et al., 2017). Sharma et al. developed a structure switching aptamer assay for AFM_1_ detection. However, the selectivity of the aptamer for AFM_1_ was not clear since only irrelevant OTA and AFB_1_ were chosen as interferences to study the cross-reaction (Sharma et al., 2016). Cross-activity tests between other toxins (especially the structural analogs AFB_1_, AFB_2_, AFG_1_, and AFG_2_) should be carried out to answer the question whether the aptasensor is suitable for quantifying the AFM_1_ concentration in real samples. In addition, the coexistence of multiple mycotoxins in foods is a common and complex phenomenon. Therefore, the effect of mix-mycotoxins on the specificity of the aptasensor should be investigated.

Thus, we developed the biosensor for the detection of AFM_1_ based on the specific aptamer, as well as the study of optimal aptamer complementary DNA and structure conversion signal. In the specificity experiment part, we detected the cross-reaction of AFB_1_, AFB_2_, AFG_1_, AFG_2_, OTA, ZEN, FB_1_, and mix-mycotoxins for the aptasensor performance. In addition, this proposed aptasensor was confirmed to be simple, fast, convenient, economical and effective. The method represents a new practical application of aptamers for the detection of AFM_1_ in milk and other dairy products.

## Materials and Methods

### Materials and Reagents

AFM_1_, AFB_1_, aflatoxin G_1_ (AFG_1_), aflatoxin G_2_ (AFG_2_), OTA, ZEN, and FB_1_ were purchased from Qingdao Pribolab Pte. Ltd. (Qingdao, China). The Tris-HCl buffer (pH 7.0) used in this study contained 10 mM 2-amino-2-(hydroxymethyl)-1,3-propanediol (Tris), 120 mM sodium chloride (NaCl), 5 mM potassium chloride (KCl), and 20 mM anhydrous calcium chloride (CaCl_2_). All chemical reagents were obtained from Shanghai Chemical Reagent Company (Shanghai, China). Phosphate-buffered saline (PBS) was obtained from Thermo Fisher Scientific (Waltham, MA, USA). Liquid whole milk samples were purchased from a local supermarket (Mengniu Dairy, Beijing, China). Distilled water was purified using a Milli-Q purification system. Measurement of fluorescence was performed in Tris-HCl buffer. AFM_1_-aptamer labeled with fluorescein (FAM) and its complementary sequences labeled with carboxytetramethylrhodamine (TAMRA) were synthesized by Wuhan GeneCreate Biological Engineering (Wuhan, China) and purified by HPLC. Their sequences were as follows:

AFM_1_ Aptamer (Malhotra et al., 2014):

5′-ATC CGT CAC ACC TGC TCT GAC GCT GGG GTC GAC CCG GAG AAA TGC ATT CCC CTG TGG TGT TGG CTC CCG TAT-FAM-3′

Complementary DNA (cDNA):

cDNA1: 5′-TAMRA- ATA CGG GA-3′
cDNA2: 5′-TAMRA- ATA CGG GAG -3′
cDNA3: 5′-TAMRA- ATA CGG GAG C-3′
cDNA4: 5′-TAMRA- ATA CGG GAG CC-3′
cDNA5: 5′-TAMRA- ATA CGG GAG CCA-3′
cDNA6: 5′-TAMRA- ATA CGG GAG CCA A-3′
cDNA7: 5′-TAMRA- ATA CGG GAG CCA AC-3′

### Aptamer-Based Fluorescence-Quenching Assay for AFM_1_ Detection

First, the AFM_1_ aptamer and cDNA were dissolved and diluted in Tris-HCl buffer, and 500 μL AFM_1_ aptamers (10 nM) were mixed with 500 μL cDNA. To optimization, the ratios of AFM_1_ aptamer: cDNA were set at 1:1, 1:2, 1:3, 1:4, or 1:5 (unless otherwise stated, experiments used a molar ratio of 1:2). Then, the mixture of AFM_1_ aptamer and cDNA was heated at 88°C for 5 min and allowed to stand at room temperature for at least 30 min. Next, 500 μL of different concentrations of AFM_1_ standard solution was added to the mixture while vortexing. The final reaction volume was 1.5 mL. Similarly, 500 μL Tris-HCl buffer was added to the mixture of AFM_1_ aptamer and cDNA while vortexing without addition of AFM_1_ and then the fluorescence intensity of fluorescence quenching was measured. Fluorescence intensities at excitation/emission wavelengths of 495/520 nm were recorded on a F-7000 fluorescence spectrophotometer (Hitachi, Japan). To avoid the influence of aflatoxin background fluorescence signal, we performed background correction by measuring the fluorescence of a control containing AFM_1_ and buffer without FAM-modified AFM_1_ aptamer or cDNA (Chen et al., 2017).

### Specificity Analysis

To evaluate the ability of this assay to detect AFM_1_ with high specificity, seven mycotoxins structurally related to AFM_1_ (AFB_1_, AFB_2_, AFG_1_, AFG_2_, OTA, ZEN, and FB_1_) were selected for testing. All mycotoxins were used at the same concentration (40 ng/mL). All other detection conditions were identical to those used in the AFM_1_ procedure, allowing comparison of fluorescence intensity for all toxins texted.

### Real Sample Analysis

The assay was validated for quantitative detection of AFM_1_ in liquid milk samples that were spiked with known concentrations of AFM_1_. When the concentration of AFM_1_ in milk samples was in the range of 1–100 ng/mL, the following pretreatment methods were used. Samples were spiked with AFM_1_ at a concentration of 0.5, 5, 10, or 20 ng/mL. Each sample was accurately weighed (0.5 mL) and adding into 10 mL centrifuge tubes, and then 2.5 mL of 70% methanol in water was added to extract AFM_1_ from the sample. The entire mixture was vortexed for 5 min on a Vortex-Genie (Scientific Industries, Bohemia, NY, United States), and then centrifuged at 10,000 × rpm for 10 min. The supernatant was collected, filtered through a 0.22-μm filter, and concentrated to 0.5 mL using a N1-Automatic Nitrogen Concentrator (Preekem Scientific, Shanghai, China). Finally, each of the residues was re-dissolved in 2 mL of aqueous methanol solution (5%, v/v) and stored at 4°C until needed. Five replicates were measured for each sample to assess the accuracy of the assay.

When the concentration of AFM_1_ in milk samples was in the range of 0–1.0 ng/mL, samples were pretreated to increase sample quantity. The samples accurately weighed (25 mL), spiked with AFM_1_ at a concentration of 0.5 ng/mL, heated to approximately 35–37°C, and then centrifuged at 4,000 rpm for 10 min. A 25-mL volume of milk sample was passed through the AFLAPREP^®^ M column (Biopharm, Germany) by gravity flow to facilitate toxin binding. The column was washed with 20 mL of PBS at a flow rate of approximately 5 mL per min. Toxin was eluted from the column with 1.25 mL of methanol:acetonitrile (40:60 v/v) at a flow rate of one drop per second; following elution, 1.25 mL of water was run through the column and collected in the same vial to yield a total volume of 2.5 mL, which was concentrated to 1 mL in a N1-Automatic Nitrogen Concentrator. After concentration, 1.5 mL Tris-HCl buffer was added. Subsequently, the fluorescence signal was determined. Five replicates of each sample were measured to evaluate the accuracy of the method.

## Results and Discussion

### Design of the Aptamer-Based Fluorescence Quenching Platform

**FIGURE 1:**
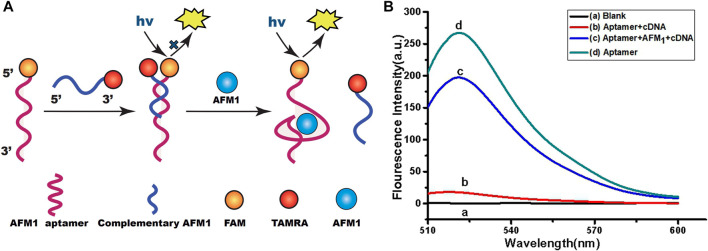
**(A)** Schematic diagram of the aptamer-based fluorescence quenching platform for the detection of AFM_1_. **(B)** Fluorescence emission spectra of the aptamer-based sensing system: blank (10 mM Tris-HCl buffer, pH 7.0) **(A)**, AFM_1_ aptamer hybridized with cDNA **(B)**, 150 ng/mL AFM_1_ **(C)**, and AFM_1_ aptamer alone **(D)**. Excitation/emission λ_ex_/λ_em_ = 495/520 nm. Reaction conditions: 10 nM AFM_1_ aptamer and 20 nM cDNA in Tris-HCl buffer (10 mM, pH 7.0).

A schematic diagram of the fluorimetric design for AFM_1_ detection is shown in Figure 1A. Theoretically, the sensor functionality was based on changes in the conformation of the aptamer after formation of the AFM_1_/aptamer complex and the resultant change in fluorescence signal. In our sensor system, AFM_1_ aptamers were modified with FAM, and 7-mer cDNAs with the TAMRA quenching group. In the absence of AFM_1_, the aptamers were hybridized with cDNA, resulting in quenching of their fluorescence due to the proximity of TAMRA-cDNA (Sharma et al., 2016). Upon the AFM_1_ addition, a structural switch was induced in the AFM_1_ aptamer, leading to formation of an AFM_1_/aptamer complex (Seok et al., 2015). Changes in the structure of the aptamer caused the release of cDNA from the aptamer, and ultimately to the recovery of aptamer fluorescence. Therefore, the concentration of AFM_1_ and the change in fluorescence intensity after the reaction were positively correlated, and the fluorescence change could be used to quantify the level of AFM_1_. To confirm that AFM_1_ could dissociate the aptamer–cDNA complex and restore the aptamer fluorescence, we added 150 ng/mL AFM_1_ to 10 nM aptamer and 20 nM cDNA in Tris-HCl buffer. Figure 1B shows that fluorescence intensity increased at least 11-fold after the addition of AFM_1_. Moreover, these findings confirm that the covalently labeled fluorophore (FAM) did not disrupt the original recognition properties of the AFM_1_ aptamer.

### Optimization of the cDNA Sequence

**FIGURE 2:**
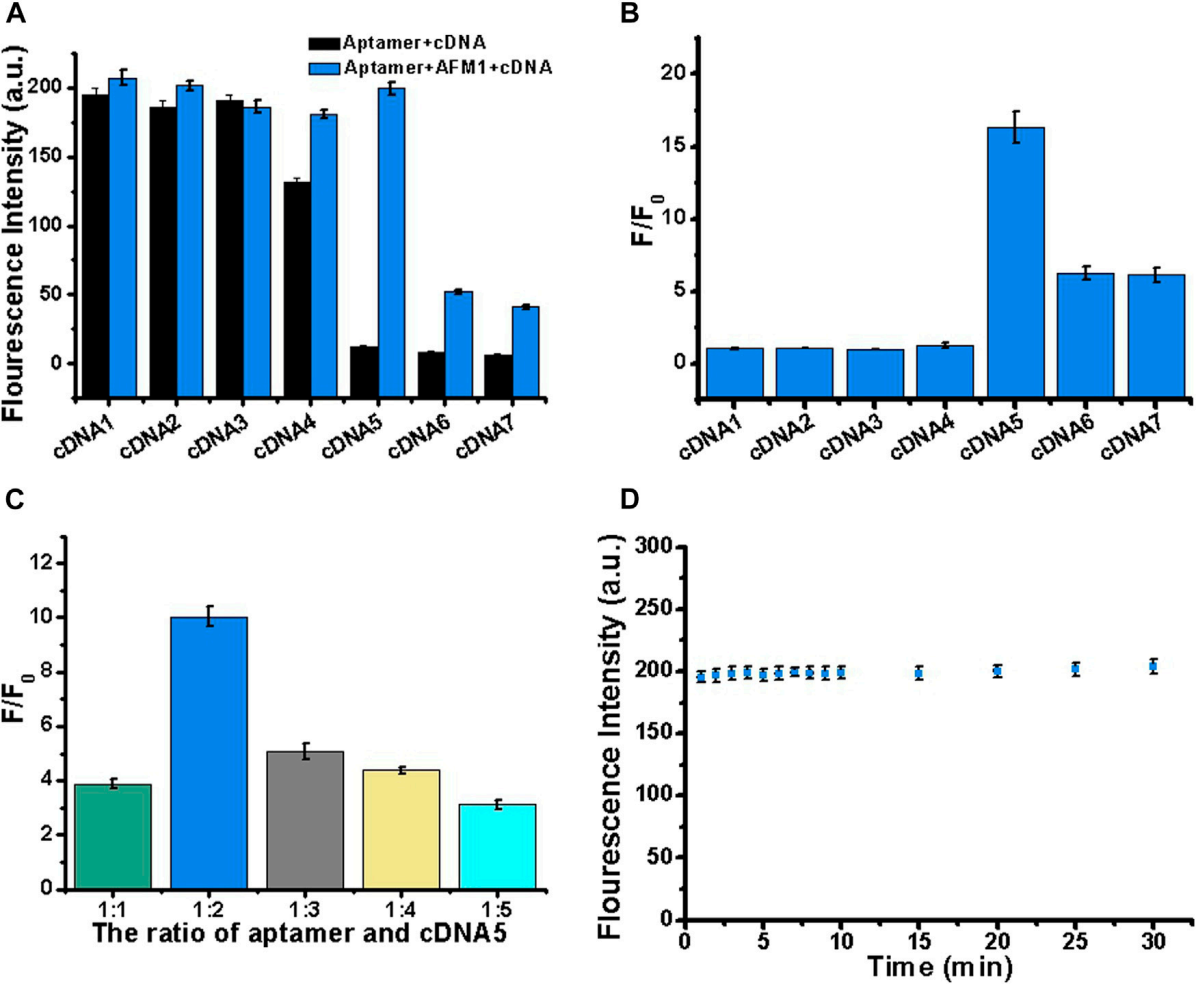
Optimization of cDNA sequence, concentration, and reaction stability. **(A)** Optimization of cDNA by varying its length (cDNAs 1–7). Black bars represent fluorescence intensity before addition of AFM_1_, and blue bars represent the fluorescence intensity after addition of 150 ng/mL AFM_1_. **(B)** Fold increase in fluorescence (F/F_0_) after addition of 150 ng/mL AFM_1_. **(C)** Optimization of the aptamer:cDNA5 concentration ratio in the presence of 150 ng/mL AFM_1_. **(D)** Study on the stability of AFM_1_ fluorescence sensor after addition of 150 ng/mL AFM_1_. Data represent means and standard deviations from three parallel experiments. Excitation/emission: λ_ex_/λ_em_ = 495/520 nm. Reaction conditions: 10 nM AFM_1_ aptamer and 20 nM cDNA in Tris-HCl buffer (10 mM, pH 7.0).

Next, we optimized the number of cDNA bases to stabilize the aptamer/cDNA duplex and minimize background fluorescence in the absence of AFM_1_. Accordingly, to optimize the sensor, we designed a series of complementary single-stranded DNA sequences. In this experiment, 10 nM AFM_1_ aptamer was added to 20 nM of cDNAs 1–7, incubated for 5 min at 88°C, and then cooled to room temperature for 30 min to *prepare* add 150 ng/mL AFM_1_. In Figure 2A, black columns represent fluorescence background after hybridization of AFM_1_ aptamers with cDNA1–cDNA7 in the absence of AFM_1_, and the blue columns represent fluorescence intensity after addition of 150 ng/mL AFM_1_. The results revealed that cDNA5–7 could effectively quench AFM_1_ aptamer fluorescence and decrease background signal in the absence of AFM_1_. Due to the short lengths of cDNAs 1–4, hybridization with *AFM1* aptamer was unstable, resulting in a higher background signal. When cDNA5 was hybridized with the aptamer, a significant increase in fluorescence (F/F_0_) was observed after addition of AFM_1_, whereas the longer cDNA sequences (cDNA6 and cDNA7) effectively quenched fluorescence (Figure 2B). This may be because of cDNA5, which contains more nucleotides than the shorter sequences, and could promote the formation of cDNA–aptamer duplexes while limiting AFM_1_-induced structural transitions of the aptamer. Therefore, we considered cDNA5 to be the optimal single-stranded cDNA sequence, and used it in subsequent experiments.

### Optimization of cDNA5 Concentration

To further improve the performance of the sensor, we optimized the cDNA5 concentration. In this experiment, 10 nM aptamer was mixed with cDNA5 at a molar ratio of 1:1, 1:2, 1:3, 1:4, or 1:5. Subsequently, 150 ng/mL AFM_1_ was added, and the change in fluorescence signal was monitored. As shown in Figure 2C, the maximum fluorescence enhancement (F/F_0_) was highest at a cDNA5 concentration of 20 nM. Thus, even at higher concentrations of cDNA5, the formation of stable cDNA5/aptamer duplexes generated low background fluorescence. When the concentration was higher than 20 nM (twice the AFM_1_ aptamer concentration), it affected the optimal AFM_1_ interaction, thereby limiting the increase in fluorescence. Accordingly, for subsequent sensing experiments, we determined the optimum concentration of cDNA5 to be 20 nM.

### Stability of the Reaction

To generate the desired fluorescence signal response, we analyzed the reaction time of AFM_1_ with the aptamer. As shown in Figure 2D, the aptamer and AFM_1_ combined rapidly: the concentration of AFM_1_ was detected at 1 min, and the fluorescence value remained stable for up to 30 min. Therefore, the fluorescence sensor had acceptable stability. We determined that a reaction time of 15 min was optimal for this assay.

### Quantitative Determination of AFM_1_

**FIGURE 3:**
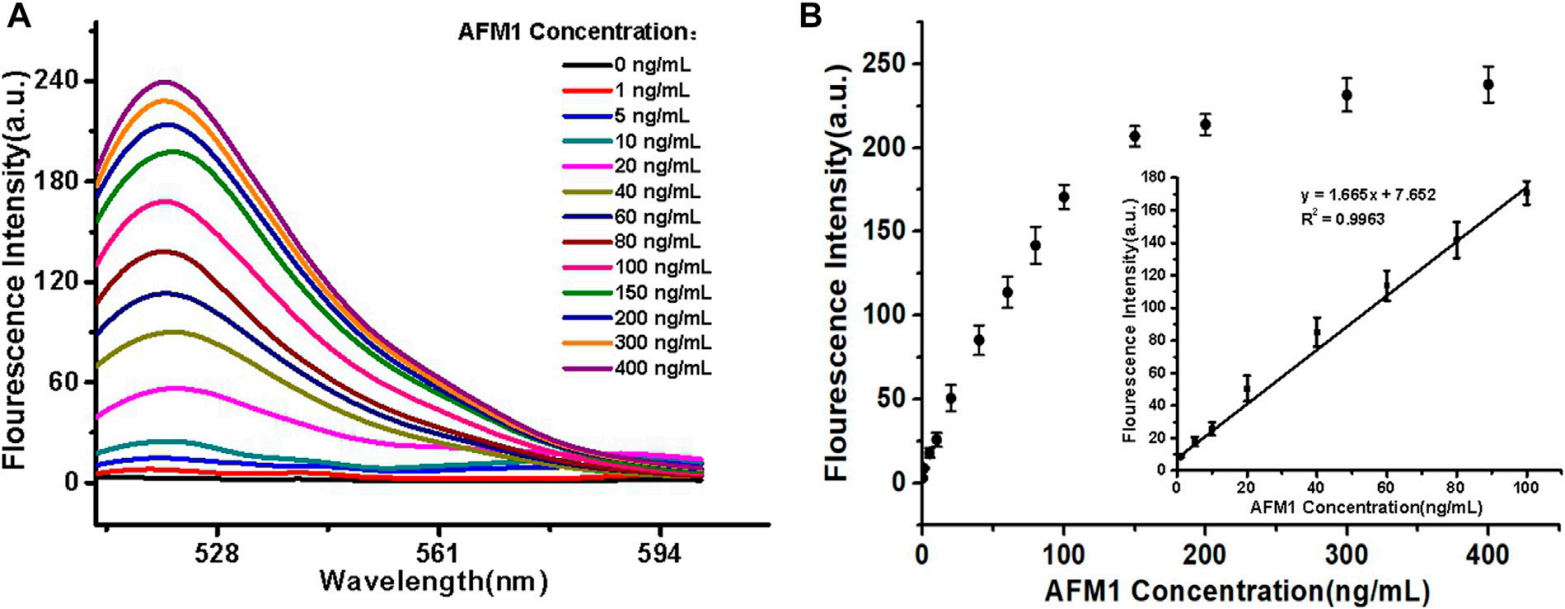
**(A)** Fluorescence emission spectra of the aptasensor. **(B)** Linear relationship between fluorescence intensity and AFM_1_ concentration. Data represent means and standard deviations from three parallel experiments. Excitation/emission: λ_ex_/λ_em_ = 495/520 nm. Reaction conditions: 10 nM AFM_1_ aptamer and 20 nM cDNA in Tris-HCl buffer (10 mM, pH 7.0).

For all experimental conditions, the excitation and emission wavelengths were 495 and 520 nm, respectively. The sensor detected different concentrations of AFM_1_ (0, 1, 5, 10, 20, 40, 60, 80, 100, 150, 200, 300, and 400 ng/mL) with corresponding fluorescence signal intensities. As the concentrations of AFM_1_ in the reaction system were increased, the released amounts of cDNA were also increased, leading to quenching groups located away from the fluorophore and an increase in fluorescence value. As shown in Figure 3A, the fluorescence intensity increased with the increased AFM_1_ concentration, and fluorescence intensity was maximal at an AFM_1_ concentration of 150 ng/mL. The concentration of AFM_1_ was linear in the range of 1–100 ng/mL (Figure 3B), and the linear regression equation was F = 1.665C + 7.652 (*R* ^2^ = 0.9963), in which F denotes the fluorescence intensity and C denotes the AFM_1_ concentration. The limit of detection (LOD) was 0.5 ng/mL with a signal-to-noize ratio of 3 (S/N = 3). Although the proposed detection method gave lower or comparable detection limits compared to other instruments and rapid screening methods (Table 1), this sensor provides a novel, simple, inexpensive and portable idea for detecting AFM_1_.

**TABLE 1:** Comparison of the aptasensor with previously reported methods for detection of AFM_1_.

Detection methods	Transduction principle	LOD	References
Instrumental methods	HPLC	6 × 10^−3^ ng/mL	Wang et al. (2012)
	SPE-UPLC–MS/MS	0.3 × 10^−3^ ng/mL	Wang and Li (2015)
Antibody-based methods	Impedimetric biosensor	1 ng/mL	Dinakaya et al. (2011)
	Enzyme immunoassay	5.0 × 10^−3^ ng/mL	Anfossi et al. (2008)
	Electrochemical immunosensors	1.0 × 10^−6^ ng/mL	Neagu et al. (2009)
Aptamer-based methods	Immunochromatographic assay	0.05 ng/mL	Wu et al. (2016)
	Label free polyaniline based aptasensor	1.98 × 10^−3^ ng/mL	Nguyen et al. (2013)
	Electrochemical impedance spectroscopy aptasensor	1.15 × 10^−3^ ng/mL	Istamboulia et al. (2016)
	RT-qPCR based aptasensor	0.03 × 10^−3^ ng/mL	Guo et al. (2016)
	Structure switching signaling aptamer assay	5.0 × 10^−3^ ng/mL	Sharma et al. (2016)
	Visual electrochemiluminescence biosensing	0.05 ng/mL	Khoshfetrat et al. (2017)
	Aptamer-based fluorescence-quenching assay	0.5 ng/mL	This work

### Specificity of the Assay

**FIGURE 4:**
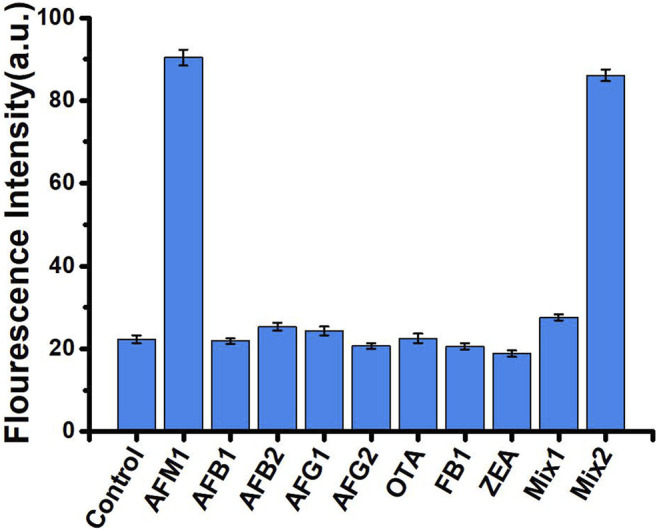
Fluorescence intensity in the absence (control) and presence of 40 ng/mL mycotoxins: AFM_1_, AFB_1_, AFB_2_, AFG_1_, AFG_2_, OTA, ZEN, and FB_1_, MIX1 (AFB_1_, AFB_2_, AFG_1_, AFG_2_, OTA, ZEN, and FB_1_), and MIX2 (AFM_1_, AFB_1_, AFB_2_, AFG_1_, AFG_2_, OTA, ZEN, and FB_1_). Excitation/emission λ_ex_/λ_em_ = 495/520 nm. Reaction conditions: 10 nM AFM1 aptamer and 20 nm cDNA in Tris-HCl buffer (10 mM, pH 7.0).

Specificity is an important measure of an aptamer sensor. To evaluate the specificity of our aptasensors, we selected seven mycotoxins with structural similarities to AFM_1_ (AFB_1_, AFB_2_, AFG_1_, AFG_2_, OTA, ZEN, and FB_1_) as controls. Each of these mycotoxins was examined at the same concentration (40 ng/mL) under the same experimental conditions used for AFM_1_. As shown in Figure 4, neither the individual mycotoxins nor a mixture of all compounds other than AFM_1_ (MIX1) significantly altered fluorescence relative to the no-mycotoxin control (*p* > 0.05). A mixture of all toxins including AFM_1_ (MIX2) had a slightly weaker effect than AFM_1_ individually, but the difference was not significant. These results confirmed that this fluorescence biosensor did not react mycotoxins other than AFM_1_, indicating that the method was highly specific for detection of AFM_1_.

### Milk Sample Analysis

To further verify practicality and feasibility, we applied our method to the detection of AFM_1_ in two different brands of spiked milk samples. Four different concentrations of AFM_1_ (0.5, 5, 10, and 20 ng/mL) were spiked into milk samples, and sample preparation was performed as described in *Real Sample Analysis* section, and five replicates were tested for each concentration. As shown in Table 2, the recoveries of the spiked milk samples were in the range 93.4–101.3% (n = 5), respectively, indicating that the aptasensor could be used as a quantitative method for AFM_1_ detection in real milk samples.

**TABLE 2:** Determination of AFM_1_ spiked into milk samples (n = 5).

Spiked concentration (ng/mL)	Detected concentrations (ng/mL)^a^	RSD (%)	Recovery (%)
0.5	0.47	4.8	93.4
5	5.1	3.6	101.3
10	9.7	2.9	97.5
20	19.4	2.6	97

^a^Mean of five replicates.

## Conclusion

In summary, we successfully developed an aptamer-based fluorescence quenching assay for direct detection of AFM_1_ and confirmed the feasibility of this method for use on milk samples. Under optimal conditions, the aptasensor exhibited linear increases in fluorescence intensity in response to increasing concentrations of AFM_1_ from 1 to 100 ng/mL, with a LOD of 0.5 ng/mL. This assay was highly specific for AFM_1_ and did not detect other toxins with similar structures, including AFB_1_, AFB_2_, AFG_1_, AFG_2_, OTA, ZEA, and FB_1_. Recoveries of AFM_1_ from spiked milk samples were in the range of 93.4–101.3% (n = 5). This assay offers a novel, simple, rapid, specific, convenient, and cost-effective approach for detection of AFM_1_, and provides a means of quantitatively determining mycotoxins in dairy products. In future studies, the method of target recycling of signal amplification to improve the sensitivity of aptamer-based fluorescence biosensors will be conducted. At the same time, we clearly understand that the interference from the milk matrix to optical measurement is typically very challenging. Therefore, strict sample pre-treatment was required for eliminating the effects of the background due to milk on the aptasensor detection, which is also the challenge and future development trend of nucleic acid aptamer detection of mycotoxins.

## Data Availability

The raw data supporting the conclusions of this article will be made available by the authors, without undue reservation.

## References

[B2] Abera B. D. Falco A. Ibba P. Cantarella G. Petti L. Lugli P. (2019). Development of flexible dispense-printed electrochemical immunosensor for aflatoxin M1 detection in milk. Sensors 19, 3912. 10.3390/s19183912 PMC6766799 31514303

[B3] Anfossi L. Calderara M. Baggiani C. Giovannoli C. Arletti E. Giraudi G. (2008). Development and application of solvent-free extraction for the detection of aflatoxin M1 in dairy products by enzyme immunoassay. J. Agric. Food Chem. 56, 1852–1857. 10.1021/jf073133d 18275143

[B4] Beitollahi H. Tajik S. Dourandish Z. Zhang K. Le Q. V. Jang H. W. (2020). Recent advances in the aptamer-based electrochemical biosensors for detecting aflatoxin B1 and its pertinent metabolite aflatoxin M1. Sensors 20, 3256. 10.3390/s20113256 PMC7309004 32521629

[B5] Busman M. Bobell J. R. Maragos C. M. (2015). Determination of the aflatoxin M1 (AFM1) from milk by direct analysis in real time - mass spectrometry (DART-MS). Food Control 47, 592–598. 10.1016/j.foodcont.2014.08.003 24588621

[B6] Chen L. Wen F. Li M. Guo X. Li S. Zheng N. (2017). A simple aptamer-based fluorescent assay for the detection of Aflatoxin B1 in infant rice cereal. Food Chem. 215, 377–382. 10.1016/j.foodchem.2016.07.148 27542489

[B7] Dinakaya E. Kinik O. Sezginturk M. Altug C. Akkoca A. (2011). Development of an impedimetric aflatoxin M1 biosensor based on a DNA probe and gold nanoparticles. Biosens. Bioelectron. 26, 3806–3811. 10.1016/j.bios.2011.02.038 21420290

[B8] Eivazzadeh K. R. Pashazadeh P. Hejazi M. Guardia M. Mokhtarzadeh A. (2016). Recent advances in Nanomaterial-mediated Bio and immune sensors for detection of Aflatoxin in food products. Trac Trends Anal. Chem. 87, 112–128. 10.1016/j.trac.2016.12.003

[B1] European Commission (2006). Commission Regulation No. 1881/2006/EC of 19 December 2006 setting maximum levels for certain contaminants in foodstuffs. Off. J. Eur. Union L. 364, 5–24.

[B9] Guo X. Wen F. Zheng N. Li S. Fauconnier M.-L. Wang J. (2016). A qPCR aptasensor for sensitive detection of aflatoxin M1. Anal. Bioanal. Chem. 408, 5577–5584. 10.1007/s00216-016-9656-z 27334718

[B10] Iqbal S. Z. Jinap S. Pirouz A. A. Ahmad Faizal A. R. (2015). Aflatoxin M1 in milk and dairy products, occurrence and recent challenges: a review. Trends Food Sci. Technol. 46, 110–119. 10.1016/j.tifs.2015.08.005

[B11] Istamboulia G. Paniel N. Zara L. Reguillo G. L. Barthelmebs L. Nogue T. (2016). Development of an impedimetric aptasensor for the determination of aflatoxin M1 in milk. Talanta 146, 464–469. 10.1016/j.talanta.2015.09.012 26695291

[B12] Karczmarczyk A. Dubiak-Szepietowska M. Vorobii M. Rodriguez-Emmenegger C. Dostálek J. Feller K.-H. (2016). Sensitive and rapid detection of aflatoxin M1 in milk utilizing enhanced SPR and p(HEMA) brushes. Biosens. Bioelectron. 81, 159–165. 10.1016/j.bios.2016.02.061 26945182

[B13] Kav K. Col R. Kaan Tekinsen K. (2011). Detection of aflatoxin M1 levels by ELISA in white-brined Urfa cheese consumed in Turkey. Food Control 22, 1883–1886. 10.1016/j.foodcont.2011.04.030

[B14] Khoshfetrat S. M. Bagheri H. Mehrgardi M. A. (2017). Visual electrochemiluminescence biosensing of aflatoxin M1 based on luminol-functionalized, silver nanoparticle-decorated graphene oxide. Biosens. Bioelectron. 100, 382–388. 10.1016/j.bios.2017.09.035 28950248

[B15] Kordasht H. K. Moosavy M. H. Hasanzadeh M. Soleymani J. Mokhtarzadeh A. (2019). Determination of aflatoxin M1 using aptamer based biosensor on the surface of dendritic fibrous nano-silica functionalized by amine groups. Anal. Methods 11, 3910–3919. 10.1039/C9AY01185D 35345244

[B16] Li P. Zhang Q. Zhang W. Zhang J. Chen X. Jiang J. (2009). Development of a class-specific monoclonal antibody-based ELISA for aflatoxins in peanut. Food Chem. 115, 313–317. 10.1016/j.foodchem.2008.11.052

[B17] Li S. Min L. Wang P. Zhang Y. Zheng N. Wang J. (2017). Aflatoxin M1 contamination in raw milk from major milk-producing areas of China during four seasons of 2016. Food Control 82, 121–125. 10.1016/j.foodcont.2017.06.036

[B37] Li X. Li P. Zhang Q. Li Y. Zhang W. Ding X. (2012). Molecular characterization of monoclonal antibodies against aflatoxins: a possible explanation for the highest sensitivity. Anal. Chem. 84, 5229–5235. 10.1021/ac202747u 22548609

[B18] Liu D. Huang Y. Wang S. Liu K. Chen M. Xiong Y. (2015). A modified lateral flow immunoassay for the detection of trace aflatoxin M1 based on immunomagnetic nanobeads with different antibody concentrations. Food Control 51, 218–224. 10.1016/j.foodcont.2014.11.036

[B19] Malhotra S. Pandey A. K. Rajput Y. S. Sharma R. (2014). Selection of aptamers for aflatoxin M1 and their characterization. J. Mol. Recognit. 27, 493–500. 10.1002/jmr.2370 24984866

[B20] Mao J. Lei S. Liu Y. Xiao D. Fu C. Zhong L. (2015). Quantification of aflatoxin M1 in raw milk by a core-shell column on a conventional HPLC with large volume injection and step gradient elution. Food Control 51, 156–162. 10.1016/j.foodcont.2014.11.022

[B21] Neagu D. Perrino S. Micheli L. Palleschi G. Moscone D. (2009). Aflatoxin M1 determination and stability study in milk samples using a screen-printed 96-well electrochemical microplate. Int. Dairy J. 19, 753–758. 10.1016/j.idairyj.2009.06.004

[B22] Nguyen B. H. Tran L. D. Do Q. P. Nguyen H. L. Tran N. H. Nguyen P. X. (2013). Label-free detection of aflatoxin M1 with electrochemical Fe3O4/polyaniline-based aptasensor. Mater. Sci. Eng. C 33, 2229–2234. 10.1016/j.msec.2013.01.044 23498252

[B23] Parker C. O. Tothill I. E. (2009). Development of an electrochemical immunosensor for aflatoxin M1 in milk with focus on matrix interference. Biosens. Bioelectron. 24, 2452–2457. 10.1016/j.bios.2008.12.021 19167207

[B24] Pietri A. Fortunati P. Mulazzi A. Bertuzzi T. (2016). Enzyme-assisted extraction for the HPLC determination of aflatoxin M1 in cheese. Food Chem. 192, 235–241. 10.1016/j.foodchem.2015.07.006 26304342

[B25] Rhouati A. Catanante G. Nunes G. Hayat A. Marty J.-L. (2016). Label-Free aptasensors for the detection of mycotoxins. Sensors 16, 2178. 10.3390/s16122178 PMC5191157 27999353

[B26] Sassahara M. Pontes Netto D. Yanaka E. K. (2005). Aflatoxin occurrence in foodstuff supplied to dairy cattle and aflatoxin M1 in raw milk in the North of Paraná state. Food Chem. Toxicol. 43, 981–984. 10.1016/j.fct.2005.02.003 15811578

[B27] Selvaraj J. N. Wang Y. Zhou L. Zhao Y. Xing F. Dai X. (2015). Recent mycotoxin survey data and advanced mycotoxin detection techniques reported from China: a review. Food Addit. Contam. A 32, 440–452. 10.1080/19440049.2015.1010185 25604871

[B28] Seok Y. Byun J.-Y. Shim W.-B. Kim M.-G. (2015). A structure-switchable aptasensor for aflatoxin B1 detection based on assembly of an aptamer/split DNAzyme. Analytica Chim. Acta 886, 182–187. 10.1016/j.aca.2015.05.041 26320651

[B29] Sharma A. Catanante G. Hayat A. Istamboulie G. Ben Rejeb I. Bhand S. (2016). Development of structure switching aptamer assay for detection of aflatoxin M1 in milk sample. Talanta 158, 35–41. 10.1016/j.talanta.2016.05.043 27343575

[B30] Shen X. Chen J. Li X. Lei H. Xu Z. Liu Y. (2017). Monoclonal antibody-based homogeneous immunoassay for three banned agonists and molecular modeling insight. Food Agric. Immunol. 28, 1438–1449. 10.1080/09540105.2017.1347149

[B31] Song S. Liu N. Zhao Z. Njumbe Ediage E. Wu S. Sun C. (2014). Multiplex lateral flow immunoassay for mycotoxin determination. Anal. Chem. 86, 4995–5001. 10.1021/ac500540z 24745689

[B32] Vaclavik L. Zachariasova M. Hrbek V. Hajslova J. (2010). Analysis of multiple mycotoxins in cereals under ambient conditions using direct analysis in real time (DART) ionization coupled to high resolution mass spectrometry. Talanta 82, 1950–1957. 10.1016/j.talanta.2010.08.029 20875601

[B33] Vdovenko M. M. Lu C.-C. Yu F.-Y. Sakharov I. Y. (2014). Development of ultrasensitive direct chemiluminescent enzyme immunoassay for determination of aflatoxin M1 in milk. Food Chem. 158, 310–314. 10.1016/j.foodchem.2014.02.128 24731347

[B39] Wang Y. Ning B. Peng Y. Bai J. Liu M. Fan X. (2013). Application of suspension array for simultaneous detection of four different mycotoxins in corn and peanut. Biosens. Bioelectron. 41, 391–396. 10.1016/j.bios.2012.08.057 23017676

[B34] Wang X. Li P. (2015). Rapid screening of mycotoxins in liquid milk and milk powder by automated size-exclusion SPE-UPLC-MS/MS and quantification of matrix effects over the whole chromatographic run. Food Chem. 173, 897–904. 10.1016/j.foodchem.2014.10.056 25466104

[B35] Wang Y. Liu X. Xiao C. Wang Z. Wang J. Xiao H. (2012). HPLC determination of aflatoxin M1 in liquid milk and milk powder using solid phase extraction on OASIS HLB. Food Control 28, 131–134. 10.1016/j.foodcont.2012.04.037

[B36] Wu C. Liu D. Peng T. Shan S. Zhang G. Xiong Y. (2016). Development of a one-step immunochromatographic assay with two cutoff values of aflatoxin M1. Food Control 63, 11–14. 10.1016/j.foodcont.2015.11.010

[B38] Yang Y. Li W. Shen P. Liu R. Li Y. Xu J. (2017). Aptamer fluorescence signal recovery screening for multiplex mycotoxins in cereal samples based on photonic crystal microsphere suspension array. Sensors Actuators B: Chem. 248, 351–358. 10.1016/j.snb.2017.04.004

[B40] Zheng N. Li S. L. Zhang H. Min L. Gao Y. N. Wang J. Q. (2017). A survey of aflatoxin M1 of raw cow milk in China during the four seasons from 2013 to 2015. Food Control 78, 176–182. 10.1016/j.foodcont.2017.02.055

